# The role of pharmacotherapy in improving quality of life among patients with MAFLD: a systematic review and meta-analysis of randomized trials

**DOI:** 10.3389/fphar.2026.1788802

**Published:** 2026-10-19

**Authors:** Sitian Zhao, Di Meng, Chuyun Rao, Chunlan Liu, Binbin Zhang, Junping Shi

**Affiliations:** 1 College of Public Health and Nursing, Hangzhou Normal University, Hangzhou, Zhejiang, China; 2 Department of Hepatology, Affiliated Hospital of Hangzhou Normal University, Hangzhou, Zhejiang, China

**Keywords:** MAFLD, metabolic dysfunction-associated fatty liver disease (MAFLD), non-alcoholic fatty liver disease, pharmacological intervention, quality of life

## Abstract

**Background:**

Clinical studies confirm that patients with metabolic dysfunction-associated fatty liver disease (MAFLD) commonly experience a significant decline in quality of life (QOL). However, existing research findings on the efficacy of drug therapies in improving their QOL remain inconsistent, and quantitative evidence from meta-analyses is lacking. This meta-analysis aims to evaluate the effectiveness of drug therapy in improving the QOL of patients with MAFLD.

**Methods:**

We systematically searched four electronic databases from their inception to 24 October 2025. All statistical analyses were performed using Review Manager software (version 5.4).

**Results:**

The meta-analysis revealed that drug intervention had a positive effect only on the Physical Component Summary (PCS) score (SMD: 0.19, 95% CI: 0.05–0.34, I^2^ = 0, p-heterogeneity = 0.45). Subgroup analysis indicated that the ≤12-month drug intervention group demonstrated improvement in the Physical Component Summary score (SMD = 0.25, 95% CI: 0.03–0.46, I^2^ = 0, p-heterogeneity = 0.26).

**Conclusion:**

Moderate to very low-quality evidence indicates that pharmacological interventions yield a small, statistically significant improvement in the SF-36 PCS score in MAFLD patients, but this change does not meet the established minimal clinically important difference threshold for this population. To better elucidate the full impact of medications on patients’ QOL, future studies should prioritize comprehensive, multidimensional assessments of patient-reported outcomes, while remaining focused on hepatic pathology.

## 1 Introduction

Metabolic dysfunction-associated fatty liver disease (MAFLD) is a hepatic manifestation of a multisystem metabolic dysfunction-driven disorder, diagnosed by hepatic steatosis ≥5% without other liver diseases. This disease encompasses a spectrum of progressive hepatic pathological changes, evolving from simple hepatic steatosis to metabolic dysfunction-associated steatohepatitis (MASH), which involves an inflammatory response and hepatocyte injury (Cusi et al., 2022). In early 2020, an international panel of 30 experts from 22 countries issued a consensus statement proposing a new definition for MAFLD. This introduced clear and simplified diagnostic criteria, transforming NAFLD from an exclusionary to an inclusionary disease (Eslam et al., 2020). The MAFLD definition highlights the crucial role of metabolic factors in the development of fatty liver disease. A diagnosis of MAFLD is confirmed in patients withhepatic steatosis who also have either type 2 diabetes mellitus (T2DM), overweight/obesity, or at least two other metabolic risk factors. Despite the updated diagnostic criteria, studies indicate that more than 99% of NAFLD patients meet MAFLD criteria, ensuring strong consistency between existing clinical research using NAFLD standards and new studies applying MAFLD standards (Gofton et al., 2023).

Over recent decades, the prevalence of MAFLD has surged dramatically, affecting 30% of adults globally. It has become one of the most common chronic liver diseases worldwide and a leading cause of liver transplantation in North America (Le et al., 2019). Before the current approved targeted drugs became available, research has demonstrated that certain hypoglycemic agents, hepatoprotective drugs, and vitamin E possess certain clinical efficacy in improving liver inflammation and protecting hepatocyte function in patients with MAFLD (Wang et al., 2024; Vogli et al., 2023; Rong et al., 2022). In 2024, Resmetirom received Food and Drug Administration (FDA) approval as the first drug specifically targeting MASH, marking a significant breakthrough in this field (Keam, 2024).

Quality of Life (QOL) is a comprehensive assessment of an individual’s perceived state of wellbeing across physical, psychological, and social dimensions. The World Health Organization (WHO) outlines a definition of QOL: “an individual’s perception of their position in life and their relationship to their goals, expectations, standards, and concerns within the cultural context of their life.” (The World Health Organization Quality of Life assessment WHOQOL, 1995) In the evolution of modern medical paradigms, QOL has emerged as a paramount comprehensive evaluation metric. By assessing patients’ physiological functions, psychological states, and social adaptation capabilities, it holistically reveals the overall impact of disease and its treatment on individuals, thereby becoming a crucial concept and objective in medical research and practice (Haraldstad et al., 2019). In clinical research design and efficacy assessment, QOL outcomes have been established as research endpoints of significant clinical and scientific value. Their importance lies in shifting the research perspective from traditional endpoints focused solely on long-term disease prognosis and complications toward a comprehensive, holistic evaluation (Flanagan et al., 2017).

Clinical studies reveal a significant decline in MAFLD patients’ QOL, primarily manifested in fatigue, pruritus, impaired general health status, and reduced vitality (Assimakopoulos et al., 2018). However, in recent years, research on new MAFLD treatments has prioritized evaluating effects on hepatic steatosis, fibrosis progression, and hepatocellular carcinoma incidence over QOL improvements (Nakajima et al., 2021; Cheung et al., 2024; Takahashi et al., 2022; Wah Kheong et al., 2017). Furthermore, existing clinical studies show inconsistent findings regarding the role of drug therapy in improving the QOL of MAFLD patients. For example, Younossi et al. observed improvements in QOL across both the physical component summary(PCS) and worry dimension (Younossi et al., 2021); however, another study found that drug intervention did not improve the physical or mental component summaries (Sanyal et al., 2010). In light of these conflicting results, the present study aims to conduct a systematic review and meta-analysis to comprehensively evaluate the impact of drug therapy on the QOL of MAFLD patients, quantify its standardized mean effect size, and provide theoretical support and new clinical insights for therapeutic interventions.

## 2 Methods

Our systematic review and meta-analysis were conducted in accordance with the Preferred Reporting Items for Systematic Reviews and Meta-Analyses (PRISMA) statement (Page et al., 2021). Our protocol is registered on PROSPERO (CRD420251174503).

### 2.1 Search strategy

We searched PubMed, the Cochrane Central Register of Controlled Trials, Embase, and Web of Science databases for relevant trials from their inception to 24 October 2025. The search strategy was developed based on the “Population, Intervention, Comparison, and Outcome” principle. The search strategy incorporated a combination of Medical Subject Headings (MeSH) terms and free-text keywords. Key search terms included “NAFLD,” “quality of life,” “health-related quality of life,” “MAFLD,” “non-alcoholic fatty liver disease,” and “metabolic-associated fatty liver disease.” Furthermore, the reference lists of identified relevant articles were manually screened to identify additional pertinent studies. A comprehensive description of our search methodology is provided in Supplementary Table S1 within the Supplementary Material.

### 2.2 Study selection

Two reviewers independently assessed the eligibility of potentially relevant articles. The studies identified from the electronic databases were imported into a reference management software. Decisions to include or exclude studies were initially based on study titles and abstracts. Full-text articles were then retrieved and evaluated to determine final inclusion (recording reasons for exclusion). If articles cited additional research reports or published protocols, these supplementary materials were obtained to ensure comprehensive data extraction. Any disagreements between reviewers were resolved through consultation with a third, senior reviewer. Studies that met the following eligibility criteria were included:(1) randomized controlled trials (RCTs); (2) patients diagnosed with MAFLD (Younossi et al., 2024); (3) Outcome measures included assessments of QOL; (4) Participants aged ≥18 years. Exclusion criteria were: (1) Duplicate studies; (2) Non-human studies; (3) Reviews, conference abstracts, case reports, letters to the editor, or technical reports; (4) Observational studies (cross-sectional, cohort, or case-control studies); (5) Studies lacking a control group. Some of the studies included in the present review were conducted prior to the introduction of the MAFLD classification and were initially based on the NAFLD/NASH diagnostic criteria. Given that MAFLD is an inclusive diagnostic concept that incorporates metabolic risk factors, studies were deemed to meet the inclusion criteria provided that their clinical characteristics are consistent with the MAFLD framework.

### 2.3 Data extraction

Following literature retrieval, two independent authors extracted relevant data from included studies according to the inclusion criteria, with discrepancies resolved by a third reviewer. The following information was extracted: the first author’s name, publication year, study location, participants’ age and gender, intervention specifics (type, duration, dose, and route), QOL assessment tools, and mean and standard deviation (SD) changes in outcomes. If a study did not report SD changes from baseline to study completion, we calculated them using the formula proposed in the Cochrane Handbook. Additionally, following the Cochrane Handbook’s recommendations, we combined QOL data for two drugs when an article included multi-arm studies with different doses or two or more drugs (Higgins et al., 2024). After data extraction, each reviewer checked the other’s data for accuracy.

### 2.4 Quality assessment and risk of bias assessment

The two authors used the Cochrane Risk of Bias Tool two checklist to assess risk of bias and methodological quality of the studies (Sterne et al., 2019). The risk of bias in these studies was evaluated based on the following seven criteria: (1) random sequence generation; (2) allocation concealment; (3) blinding of patients and personnel; (4) blinding of outcome assessors; (5) incomplete outcome data; (6) selective reporting; (7) other biases. If two assessors disagreed after an independent assessment, a third assessor was used to facilitate consensus. The GRADE (Grades of Recommendation, Assessment, Development, and Evaluation) framework was used to evaluate the certainty of the underlying evidence (Brozek et al., 2009). According to factors that might downgrade the certainty, the system classified the certainty of evidence as high, moderate, low, or very low. These factors were risk of bias, inconsistency, imprecision, indirectness, and publication bias.

### 2.5 Statistical analysis

All analyses were conducted using Review Manager software (version 5.4). Effect sizes were calculated using the mean change from baseline for each group. Forest plots were then constructed to provide a visual summary of the results. When different scales measured the same outcome, continuous variables were pooled using standardized mean differences (SMDs). The Cochrane Q test (p-level of significance set at 0.1), along with the I^2^ value, was used to assess between-study heterogeneity. Following Cochrane Manual standards, I^2^ < 40% indicates potentially insignificant heterogeneity, 30%–60% indicates moderate heterogeneity, 50%–90% indicates substantial heterogeneity, and 75%–100% indicates considerable heterogeneity. Additionally, we planned subgroup analyses based on intervention duration and the occurrence of weight loss. Pre-specified subgroup analyses will be conducted only if each subgroup contains at least two studies. Sensitivity analyses, excluding individual studies, will be performed to assess the stability of the results. Publication bias assessment (e.g., funnel plot or Egger’s test) was not conducted due to insufficient power for testing, as fewer than 9–10 studies per questionnaire type addressed any specific outcome. Except for heterogeneity tests, all statistical tests were set at a significance level of P < 0.05.

## 3 Results

### 3.1 Search results

The details of the literature search and screening process are shown in Figure 1. Initially, 3,737 records were identified through the systematic search strategy. After excluding 1264 duplicate articles, we screened the remaining 2,473 articles and excluded 2,431 based on title and abstract review. Finally, we assessed the full text of 23 articles and excluded 12 due to incomplete data or other reasons. Consequently, 11 articles were included in our meta-analysis (Figure 1).

**FIGURE 1:**
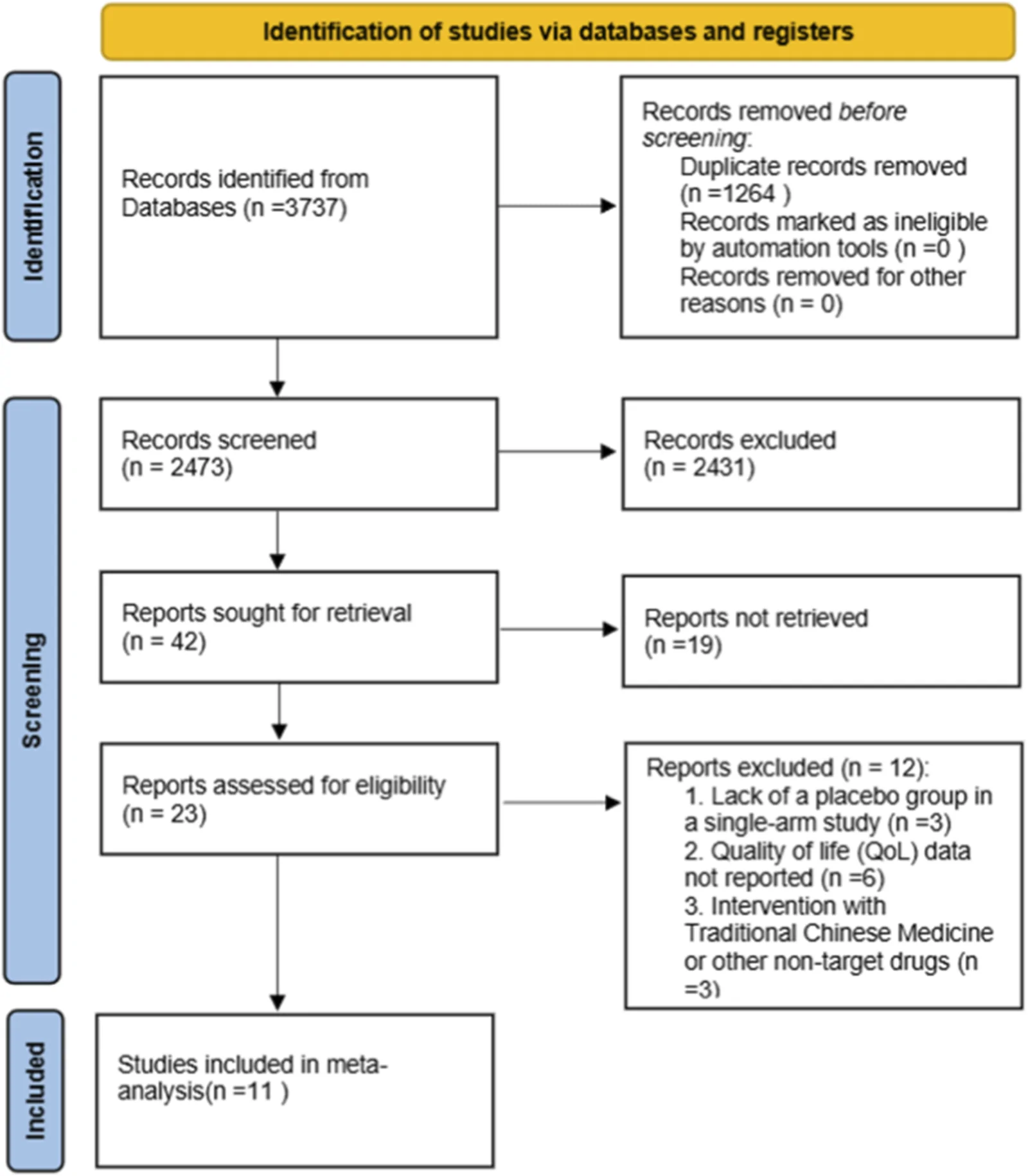
Study search and selection procedure.

### 3.2 Study characteristics

The total number of participants was 3,087, with 1,467 males (47.5%) and 1,620 females (52.5%). The duration of interventions ranged from 84 days to 96 weeks. Six studies (Pothula et al., 2022), (Neuschwander-Tetri et al., 2015), (Younossi et al., 2022a), (Armstrong et al., 2016), (Ghanbari et al., 2024), (Lin et al., 2025) employed the Short Form 36 Health Survey (SF-36), three (Younossi et al., 2022b), (El-Kady et al., 2022), (Younossi et al., 2025) used the Chronic Liver Disease Questionnaire (CLDQ), one (Kessoku et al., 2020) utilized the Short Form-8 (SF-8), and another (McPherson et al., 2017) assessed QOL using both the SF-36 and CLDQ questionnaires. A summary of the characteristics of the studies is presented in Table 1.

**TABLE 1:** Characteristics of the included randomized controlled trials. Note: CG, control group; EG, experimental group; CLDQ, chronic liver disease questionnaire; SF-8, short form-8 health survey; SF-36, short form-36 health survey.

Study	Year	Country	Age		%Male		Number of participants		Type		Dose	Duration of follow-up	Evaluation tool
			EG	CG	EG	CG	EG	CG	EG	CG	EG		
Vamsi Krishna Pothula Rajendra	2022	India	40.2 ± 10.1	36.6 ± 7.5	14 (46.7%)	16 (53.3%)	29	29	Silymarin	Placebo	320 mg	84days	SF-36
Stuart McPherson	2017	England	56 ± 13.4	46 ± 14.6	13(54%)	12 (57%)	20	21	Losartan	Placebo	50 mg	96weeks	SF-36,CLDQ
Brent A Neuschwander-Tetri	2015	USA	52 ± 11	51 ± 12	43 (30%)	53 (37%)	110	109	Obeticholic acid	Placebo	25 mg	72 weeks	SF-36
Zobair M Younossi	2022a	USA	51.8 ± 10.4	47.3 ± 11.7	38 (45%)	24 (59%)	84	41	Resmetirom	Placebo	80 mg	36 weeks	SF-36
Takaomi Kessoku	2020	Japan	54 ± 13	53 ± 15	34 (62%)	28 (62%)	55	45	Lubiprostone	Placebo	24 μg	12weeks	SF-8
			54 ± 16		32 (64%)		50		Lubiprostone		12 μg		
Zobair M Younossi	2022b	United States	54.2 ± 11.8	53.6 ± 11.7	171 (42%)	176 (43%)	404	407	Obeticholic acid	Placebo	25 mg	18months	CLDQ
			54.4 ± 11.0		177 (43%)		407		Obeticholic acid		10 mg		
Rasha R El-Kady	2022	Egypt	45.6 ± 7.3	47.6 ± 9.8	13(42%)	13(43%)	31	30	Nicotinamide	Placebo	1000 mg	12weeks	CLDQ
Matthew James Armstrong	2016	UK	50 ± 11	52 ± 12	18 (69%)	13(50%)	23	22	Liraglutide	Placebo	1.8 mg	48weeks	SF-36
Parisa Ghanbari	2024	Iran	43.52 ± 8.12	44.88 ± 10.14	10 (40%)	14 (56%)	25	25	GSE	Placebo	520 mg	2months	SF-36
Zobair M Younossi	2025	United States	57 ± 10.8	57.1 ± 10.5	141(43.7%)	143(44.5%)	323	321	Resmetirom	Placebo	100 mg	52 weeks	CLDQ-NAFLD
			55.9 ± 11.5		140(43.5%)		322		Resmetirom		80 mg		
Jiayang Lin	2025	China	34.7 ± 9.5	35.4 ± 10.9	67 (86%)	64 (84%)	78	76	Dapagliflozin	Placebo	10 mg	48 weeks	SF-36

### 3.3 Quality assessment

This meta-analysis assessed the methodological quality of the 11 included studies using the Cochrane Risk of Bias tool. All studies reported specific randomization methods, complete outcome data, and no selective reporting of results, indicating low risk of bias. However, Pothula et al. (2022) demonstrated a high risk of bias in allocation concealment and did not describe blinding of outcome assessors, potentially introducing bias. The study by El-Kady et al. (2022) exhibited performance bias because the principal investigator conducted both the randomization and enrollment. Additionally, the study by McPherson et al. (2017) was rated as having an unclear risk of bias due to the possibility of other biases. The summary of bias risk profiles is presented in Figure 2. Supplementary Table S2 shows the details of our assessment of the certainty of the evidence for each outcome, as determined using the GRADE approach. Overall, we consider the quality of the evidence to be moderate to very low.

**FIGURE 2:**
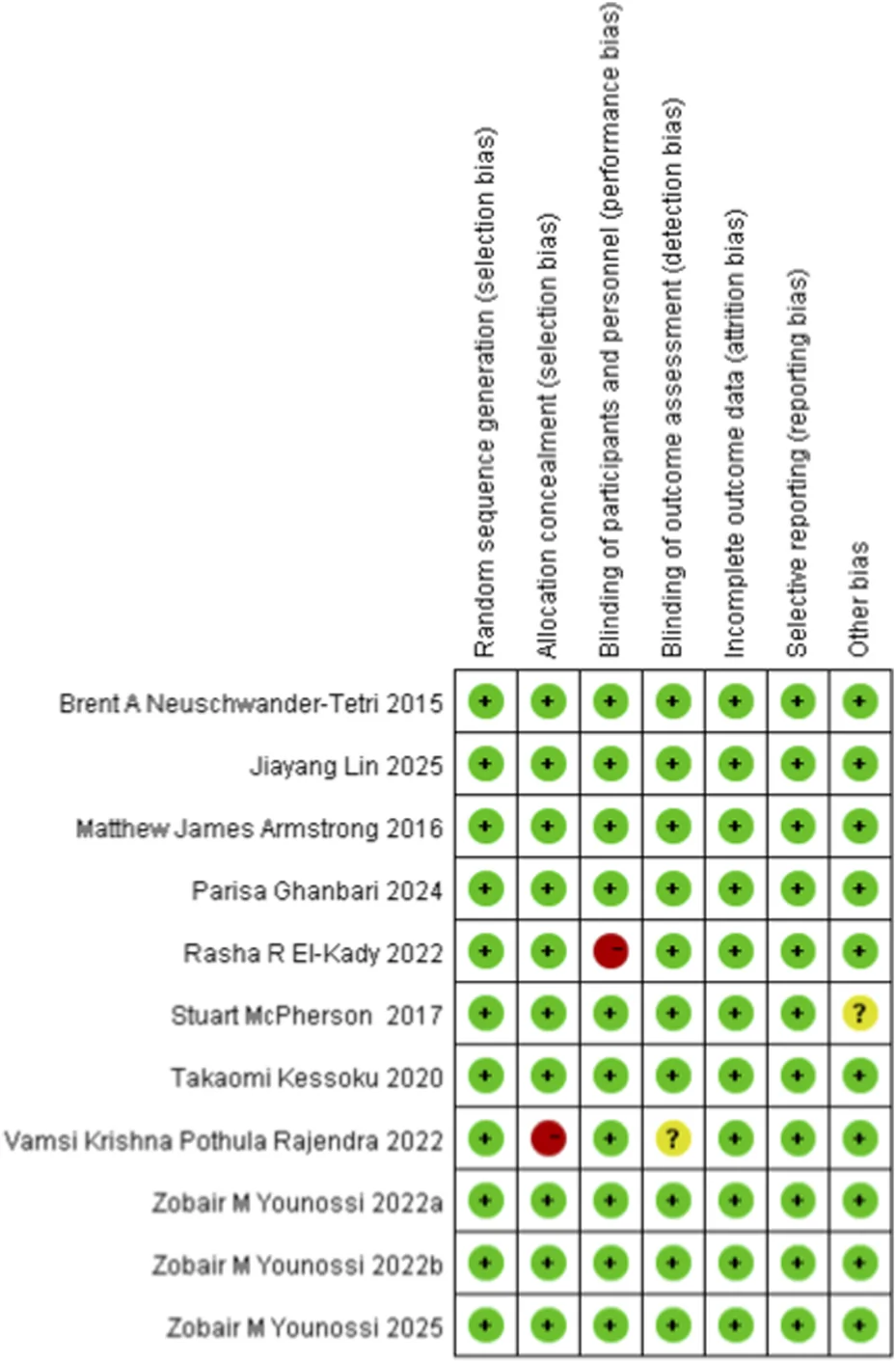
Risk of bias summary of the 11 included studies using the Cochrane Risk of Bias tool.

### 3.4 Meta-analysis

#### 3.4.1 Based on SF-36 QOL scores

SF-36 assesses QOL across eight distinct domains. These domains are aggregated into two summary scores: the PCS, calculated from physical functioning, role-physical, bodily pain, and general health; and the Mental Component Summary (MCS), calculated from vitality, social functioning, role-emotional, and mental health (Ware and Sherbourne, 1992).

Seven Studies (Armstrong et al., 2016; Neuschwander-Tetri et al., 2015; Ghanbari et al., 2024; Lin et al., 2025; McPherson et al., 2017; Kessoku et al., 2020; Younossi et al., 2022a) of PCS and MCS data were eligible for meta-analysis. The PCS scores in the drug intervention group were superior to those in the placebo group, indicating a positive effect of drug intervention on PCS improvement (SMD: 0.19, 95% CI: 0.05–0.34, I^2^ = 0, p-heterogeneity = 0.45) (Figure 3). However, drug intervention showed no significant association with MCS improvement in MAFLD patients (SMD: −0.08, 95% CI: −0.30 to 0.13, I^2^ = 50%, p-heterogeneity = 0.06) (Figure 4).

**FIGURE 3:**
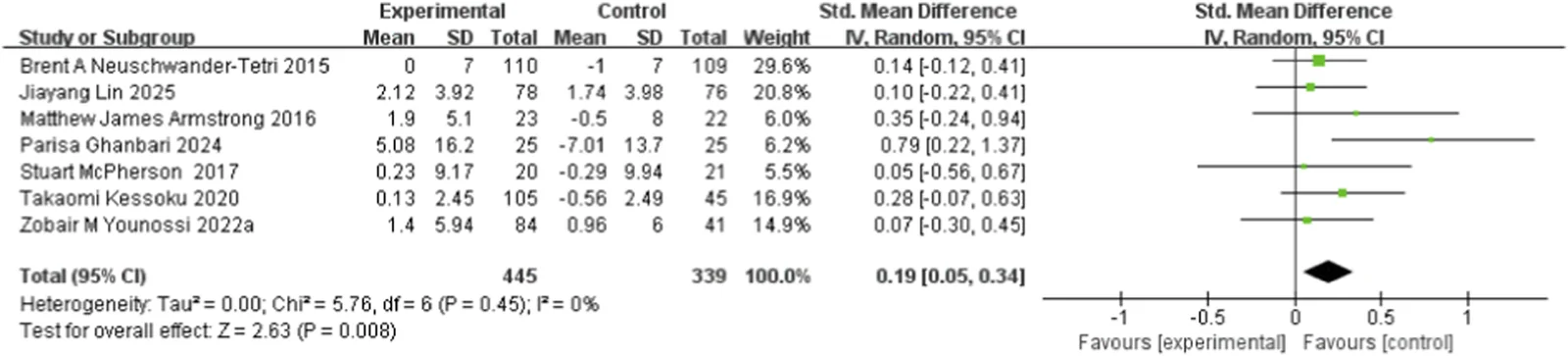
Forest plot of the effect of pharmacotherapy on the PCS of SF-36 in patients with MAFLD. Note: CI, confidence interval; IV, inverse variance; SD, standard deviation; SF-36, short form-36 health survey; PCS, physical component summary; MAFLD, metabolic dysfunction-associated fatty liver disease.

**FIGURE 4:**
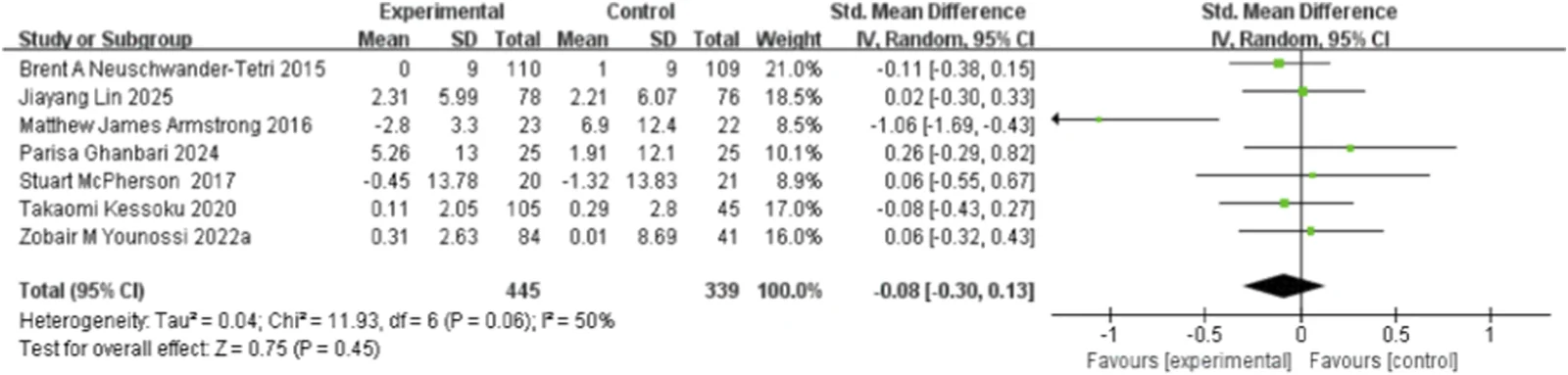
Forest plot of the effect of pharmacotherapy on the MCS of SF-36 in patients with MAFLD. Note: CI, confidence interval; IV, inverse variance; SD, standard deviation; MCS, mental component summary; SF-36, short form-36 health survey; MAFLD, metabolic dysfunction-associated fatty liver disease.

Analysis of the eight individual SF-36 domains revealed that the drug intervention did not yield statistically significant improvements in any specific QOL domain compared to the placebo group in patients with MAFLD (Supplementary Figures S1–S8).

#### 3.4.2 Based on the CLDQ QOL score

Four studies (McPherson et al., 2017; El-Kady et al., 2022; Younossi et al., 2022b; Younossi et al., 2025) reported chronic liver disease questionnaire(CLDQ)outcomes comparing drug interventions with placebo. Compared to the placebo group, drug interventions did not improve the CLDQ total score in MAFLD patients (SMD: −0.05, 95% CI: −0.14, 0.04, I^2^ = 0, p-heterogeneity = 0.76) (Figure 5).

**FIGURE 5:**
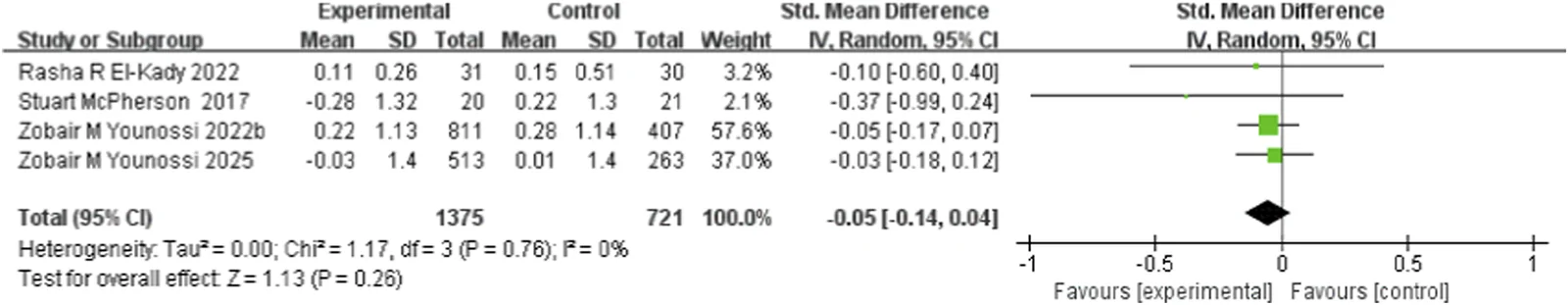
Forest plot of the effect of pharmacotherapy on the CLDQ total score in patients with MAFLD. Note: CI, confidence interval; IV, inverse variance; SD, standard deviation; CLDQ, chronic liver disease questionnaire; MAFLD, metabolic dysfunction-associated fatty liver disease.

Meta-analyses of the six CLDQ domains (Abdominal Symptoms, Fatigue, Systemic Symptoms, Activity, Emotional Functioning, and Worry) individually showed that drug interventions did not significantly improve any specific QOL domain compared with placebo (Supplementary Figures S9–S14).

### 3.5 Subgroup analysis

Due to an insufficient number of studies incorporating the CLDQ, subgroup analyses were performed only for the MCS and PCS of the SF-36.

Subgroup analysis based on intervention duration showed that in the subgroup with intervention ≤12 months (Armstrong et al., 2016; Ghanbari et al., 2024; Lin et al., 2025; Kessoku et al., 2020; Younossi et al., 2022a) drug therapy was associated with a significant improvement in PCS (SMD = 0.25, 95% CI: 0.03, 0.46; I^2^ = 0%, p-heterogeneity = 0.26). In contrast, no significant change in PCS was observed in the subgroup with intervention >12 months (Neuschwander-Tetri et al., 2015; McPherson et al., 2017) (SMD = 0.13, 95% CI: −0.12, 0.37; I^2^ = 0%, p-heterogeneity = 0.79). For MCS, no significant improvement was found in either the ≤12-month subgroup (SMD = −0.11, 95% CI: −0.43, 0.21; I^2^ = 66%, p-heterogeneity = 0.02) or the >12-month subgroup (SMD = −0.08, 95% CI: −0.33, 0.16; I^2^ = 0%, p-heterogeneity = 0.61).

Subgroup analysis based on weight change status showed that PCS did not significantly improve in either the weight-improved subgroup (Armstrong et al., 2016; Neuschwander-Tetri et al., 2015; Lin et al., 2025; Younossi et al., 2022a) (SMD = 0.13, 95% CI: −0.04, 0.30; I^2^ = 0%, p-heterogeneity = 0.88) or the weight-not-improved subgroup (SMD = 0.22, 95% CI: −0.08, 0.53; I^2^ = 0%, p-heterogeneity = 0.53). Similarly, MCS did not show significant improvement in either the weight-improved subgroup (SMD = −0.19, 95% CI: −0.53, 0.15; I^2^ = 71%, p-heterogeneity = 0.02) or the weight-not-improved subgroup (Kessoku et al., 2020; McPherson et al., 2017) (SMD = −0.04, 95% CI: −0.35, 0.26; I^2^ = 0%, p-heterogeneity = 0.70).

### 3.6 Sensitivity analyses

Sensitivity analyses indicate that after sequentially excluding individual studies, the positive effects of drug therapy on improving QOL in patients with MAFLD remained consistent in both direction and magnitude (Supplementary Figures S15–S21).

## 4 Discussion

This systematic review and meta-analysis of randomized controlled trials indicates that, following drug treatment, patients with MAFLD experienced only a slight statistical improvement in quality of life on the PCS dimension. No significant improvements were observed in MCS or specific QOL domains, such as Physical Functioning, Physical Role Functioning, Bodily Pain, General Health Perceptions, Vitality, Social Functioning, Emotional Role Functioning, Mental Health, Worry, Fatigue Symptoms, Systemic Symptoms, Activity, Emotional Functioning, and Abdominal Symptoms. Subgroup analysis suggested that intervention duration may influence patient QOL. The experimental group demonstrated superior PCS scores compared to the control group in studies of 12 months or less.

Collectively, our findings suggest that drug therapy exerts a selective, physical-focused impact on QOL in patients with MAFLD. Specifically, although the pooled analysis revealed a statistically significant improvement in PCS scores following pharmacological intervention, this beneficial change was accompanied by a small effect size (SMD = 0.19). For the SF-36, a 5 to 10 point difference in a score (when scores are scaled on 0 to 100 scale) are considered to reach the minimal clinically important difference (Desai et al., 2025). In this context, the 1–2 point PCS improvement observed in our analysis is statistically detectable but insufficient to produce tangible, patient-perceived improvements in physical function and overall quality of life. Consequently, whilst pharmacological interventions may result in measurable changes in health-related quality of life, these changes are unlikely to represent substantial, patient-perceived improvements in routine clinical practice. Future trials should incorporate responder analyses and apply clinically meaningful thresholds in order to better assess patient-centred benefits. However, no significant benefits were noted in the MCS dimension. This suggests that current pharmacological interventions may be insufficient to effectively alleviate the psychological burden experienced by patients with MAFLD. The discrepancy between improvements in physical and mental health may stem from multiple factors. First, MAFLD itself exerts domain-specific effects on QOL. Research indicates that impaired QOL in patients primarily affects somatic-related dimensions, such as overall health, vitality, and physical functioning (Sayiner et al., 2016; David et al., 2009). Meanwhile, differences in mental health domains are not significant compared to healthy controls (Golabi et al., 2016). Additionally, systematic screening and comprehensive interventions for mental health issues in MAFLD patients are inadequate in clinical practice, which may contribute to the limited efficacy of medications on MCS.

Research by Younossi et al. indicates that resmetirom treatment is associated with improvements in certain QOL scores among MAFLD patients, with these improvements persisting and intensifying over extended treatment periods (Younossi et al., 2022a). However, the meta-analysis in this study reveals that shorter intervention cycles -particularly those under 12 months - actually demonstrate more pronounced advantages in improving PCS. This discrepancy requires careful consideration. We hypothesize that this may be related to the timing of the assessments: short-term studies often evaluate outcomes at the peak of the effects of the intervention, whereas long-term studies may capture outcomes after the effects have plateaued. Therefore, the current findings do not negate the potential value of long-term interventions, but rather suggest significant heterogeneity in assessment timing across studies. Future research should standardize the frequency and timing of QOL assessments to depict their temporal trajectories more comprehensively and accurately.

Another key consideration is the heterogeneity of the pharmacological interventions included in this meta-analysis. The included drugs span different therapeutic classes, including metabolic modulators, antifibrotic agents and nutritional supplements, each with distinct mechanisms of action and therapeutic targets. Although statistical heterogeneity was low in the primary PCS analysis, differences in therapeutic mechanisms, duration of intervention, disease severity and patients’ baseline characteristics may all influence the extent of improvement in QOL. Therefore, the pooled effect size should be interpreted as reflecting the overall efficacy of pharmacological interventions, rather than as evidence supporting the superiority of any single drug.

This study found no significant correlation between weight improvement and changes in QOL in subgroup analyses, which differs from the conclusions of previous research (Tapper and Lai, 2016). One possible reason for this discrepancy is that most of the studies included in this analysis did not stratify patients based on detailed weight loss levels. Consequently, the analyzed sample contained a large number of cases where weight loss did not reach the clinically relevant threshold of 5% (Eslam et al., 2025). Multiple evidence-based medical studies indicate that weight loss has a clear threshold effect on QOL. For example, a 36-week clinical trial of resmetirom observed significant improvements in mental health domains in the subgroup that achieved ≥5% weight loss (Younossi et al., 2022a). Furthermore, Laura et al. confirmed that weight loss of approximately 5 kg (equivalent to >5% of baseline weight) was independently associated with improvements in physical functioning, overall health, and vitality scores (Heath et al., 2022). Therefore, the findings of this study do not negate the potential value of weight management in improving QOL. Rather, they suggest that future intervention studies should focus on achieving clinically meaningful weight loss. To more accurately elucidate the relationship between weight change and QOL, it is recommended that analyses stratify outcomes based on different levels of weight loss.

In summary, we recommend that drug interventions prioritize not only improving liver pathology but also optimizing metabolic markers (particularly weight management), implementing lifestyle modifications, and selecting appropriate treatment durations. This multidimensional integrated approach enhances not only clinical outcomes but also patients’ overall experience with liver disease.

## 5 Limitations

The primary strength of this meta-analysis lies in its integration of direct evidence from historical and recently published randomized controlled trials. These trials encompass multiple outcome measures and examine the impact of pharmacological interventions on the QOL of patients with MAFLD. However, several limitations warrant careful consideration. Firstly, while 11 studies were included, the use of various assessment scales, such as the CLDQ and SF-36, which differ in their item structure, prevented data pooling. Consequently, some effect sizes corresponded to fewer than five studies. The limited number of included studies meant that it was not possible to carry out a formal statistical assessment of publication bias using methods such as funnel plots or Egger’s test, which could affect the reliability of the findings. Secondly, all significant results were derived from the SF-36 scale, while the CLDQ questionnaire, which is designed to assess liver disease-specific symptoms, failed to yield statistically significant outcomes. This partially limits the comprehensiveness of the study’s conclusions.Thirdly, several included trials were conducted prior to the introduction of the MAFLD nomenclature and originally enrolled participants based on NAFLD or NASH criteria. These studies were deemed to meet the inclusion criteria because, when metabolic risk factors were taken into account, many patients with NAFLD or NASH met the current MAFLD diagnostic criteria. Although we extracted available metabolic data—including overweight or obesity, type 2 diabetes and dyslipidaemia—to assess their compatibility with the MAFLD framework as far as possible, some studies still lacked a complete metabolic phenotype. Consequently, residual diagnostic heterogeneity may exist, and this should be taken into account when interpreting the pooled estimates.

## 6 Conclusion

As modern medical models have advanced, QOL has become an important outcome measure that cannot be overlooked. This study employed a systematic review and meta-analysis approach and conducted a comprehensive literature search of RCTs. Although pharmacological interventions yielded a statistically significant improvement in the SF-36 PCS score (SMD = 0.19), the magnitude of this effect was small and did not meet the established minimal clinically important difference threshold for clinically meaningful change. Therefore, we recommend that drug interventions for MAFLD should focus not only on improving liver pathology but also on comprehensive, multidimensional intervention strategies.

## Data Availability

The original contributions presented in the study are included in the article/Supplementary Material, further inquiries can be directed to the corresponding author.

## References

[B1] Armstrong M. J. Gaunt P. Aithal G. P. Barton D. Hull D. Parker R. (2016). Liraglutide safety and efficacy in patients with non-alcoholic steatohepatitis (LEAN): a multicentre, double-blind, randomised, placebo-controlled phase 2 study. Lancet 387 (10019), 679–690. 10.1016/s0140-6736(15)00803-x 26608256

[B2] Assimakopoulos K. Karaivazoglou K. Tsermpini E.-E. Diamantopoulou G. Triantos C. (2018). Quality of life in patients with nonalcoholic fatty liver disease: a systematic review. J. Psychosomatic Res. 112, 73–80. 10.1016/j.jpsychores.2018.07.004 30097139

[B3] Brozek J. L. Akl E. A. Alonso-Coello P. Lang D. Jaeschke R. Williams J. W. (2009). Grading quality of evidence and strength of recommendations in clinical practice guidelines. Part 1 of 3. An overview of the GRADE approach and grading quality of evidence about interventions. Allergy 64 (5), 669–677. 10.1111/j.1398-9995.2009.01973.x 19210357

[B4] Cheung K. S. Ng H. Y. Hui R. W. H. Lam L. K. Mak L. Y. Ho Y. C. (2024). Effects of empagliflozin on liver fat in patients with metabolic dysfunction-associated steatotic liver disease without diabetes mellitus: a randomized, double-blind, placebo-controlled trial. Hepatology 80 (4), 916–927. 10.1097/hep.0000000000000855 38536017

[B5] Cusi K. Isaacs S. Barb D. Basu R. Caprio S. Garvey W. T. (2022). American Association of Clinical Endocrinology Clinical Practice Guideline for the diagnosis and management of Nonalcoholic Fatty liver disease in primary care and endocrinology clinical settings: Co-Sponsored by the American Association for the Study of liver diseases (AASLD). Endocr. Pract. 28 (5), 528–562. 10.1016/j.eprac.2022.03.010 35569886

[B6] David K. Kowdley K. V. Unalp A. Kanwal F. Brunt E. M. Schwimmer J. B. (2009). Quality of life in adults with nonalcoholic fatty liver disease: baseline data from the nonalcoholic steatohepatitis clinical research network. Hepatology 49 (6), 1904–1912. 10.1002/hep.22868 19434741 PMC2692572

[B7] Desai A. P. Orman E. S. Aridi T. G. Stump T. Nephew L. Ghabril M. S. (2025). Meaningful differences in patient-reported outcome measurement scores in liver disease. Hepatol. Commun. 9 (6), e0727. 10.1097/hc9.0000000000000727 40434652 PMC12122172

[B8] El-Kady R. R. Ali A. K. El Wakeel L. M. Sabri N. A. Shawki M. A. (2022). Nicotinamide supplementation in diabetic nonalcoholic fatty liver disease patients: randomized controlled trial. Ther. Adv. Chronic Dis. 13, 20406223221077958. 10.1177/20406223221077958 35222903 PMC8874180

[B9] Eslam M. Newsome P. N. Sarin S. K. Anstee Q. M. Targher G. Romero-Gomez M. (2020). A new definition for metabolic dysfunction-associated fatty liver disease: an international expert consensus statement. J. Hepatol. 73 (1), 202–209. 10.1016/j.jhep.2020.03.039 32278004

[B10] Eslam M. Fan J. G. Yu M. L. Wong V. W. Cua I. H. Liu C. J. (2025). The Asian Pacific association for the study of the liver clinical practice guidelines for the diagnosis and management of metabolic dysfunction-associated fatty liver disease. Hepatol. Int. 19 (2), 261–301. 10.1007/s12072-024-10774-3 40016576

[B11] Flanagan S. Damery S. Combes G. (2017). The effectiveness of integrated care interventions in improving patient quality of life (QoL) for patients with chronic conditions. An overview of the systematic review evidence. Health Qual. Life Outcomes 15 (1), 188. 10.1186/s12955-017-0765-y 28962570 PMC5622519

[B12] Ghanbari P. Raiesi D. Alboebadi R. Zarejavid A. Dianati M. Razmi H. (2024). The effects of grape seed extract supplementation on cardiovascular risk factors, liver enzymes and hepatic steatosis in patients with non-alcoholic fatty liver disease: a randomised, double-blind, placebo-controlled study. BMC Complement. Med. Ther. 24 (1), 192. 10.1186/s12906-024-04477-3 38755622 PMC11100156

[B13] Gofton C. Upendran Y. Zheng M. H. George J. (2023). MAFLD: how is it different from NAFLD? Clin. Mol. Hepatol. 29 (Suppl. l), S17–s31. 10.3350/cmh.2022.0367 36443926 PMC10029949

[B14] Golabi P. Otgonsuren M. Cable R. Felix S. Koenig A. Sayiner M. (2016). Non-alcoholic Fatty Liver Disease (NAFLD) is associated with impairment of Health Related Quality of Life (HRQOL). Health Qual. Life Outcomes 14, 18. 10.1186/s12955-016-0420-z 26860700 PMC4746896

[B15] Haraldstad K. Wahl A. Andenæs R. Andersen J. R. Andersen M. H. Beisland E. (2019). A systematic review of quality of life research in medicine and health sciences. Qual. Life Res. 28 (10), 2641–2650. 10.1007/s11136-019-02214-9 31187410 PMC6761255

[B16] Heath L. Aveyard P. Tomlinson J. W. Cobbold J. F. Koutoukidis D. A. (2022). Association of changes in histologic severity of nonalcoholic steatohepatitis and changes in patient-reported quality of life. Hepatol. Commun. 6 (10), 2623–2633. 10.1002/hep4.2044 35903833 PMC9512481

[B17] Higgins J. P. T. Thomas J. Chandler J. Cumpston M. Li T. Page M. J. (2024). Cochrane Handbook for Systematic Reviews of Interventions, version 6.5 (updated August 2024). Cochrane. Available from: https://www.cochrane.org/handbook.

[B18] Keam S. J. (2024). Resmetirom: first approval. Drugs 84 (6), 729–735. 10.1007/s40265-024-02045-0 38771485

[B19] Kessoku T. Imajo K. Kobayashi T. Ozaki A. Iwaki M. Honda Y. (2020). Lubiprostone in patients with non-alcoholic fatty liver disease: a randomised, double-blind, placebo-controlled, phase 2a trial. Lancet Gastroenterol. Hepatol. 5 (11), 996–1007. 10.1016/s2468-1253(20)30216-8 32805205

[B20] Le M. H. Yeo Y. H. Li X. Li J. Zou B. Wu Y. (2019). Global NAFLD prevalence: a systematic review and meta-analysis. Clin. Gastroenterol. Hepatol. 20(12):2809–17.e28. 10.1016/j.cgh.2021.12.002 34890795

[B21] Lin J. Huang Y. Xu B. Gu X. Huang J. Sun J. (2025). Effect of dapagliflozin on metabolic dysfunction-associated steatohepatitis: multicentre, double blind, randomised, placebo controlled trial. Bmj 389, e083735. 10.1136/bmj-2024-083735 40467095 PMC12135075

[B22] McPherson S. Wilkinson N. Tiniakos D. Wilkinson J. Burt A. D. McColl E. (2017). A randomised controlled trial of losartan as an anti-fibrotic agent in non-alcoholic steatohepatitis. PLoS One 12 (4), e0175717. 10.1371/journal.pone.0175717 28419124 PMC5395178

[B23] Nakajima A. Eguchi Y. Yoneda M. Imajo K. Tamaki N. Suganami H. (2021). Randomised clinical trial: pemafibrate, a novel selective peroxisome proliferator-activated receptor α modulator (SPPARMα), *versus* placebo in patients with non-alcoholic fatty liver disease. Aliment. Pharmacol. Ther. 54 (10), 1263–1277. 10.1111/apt.16596 34528723 PMC9292296

[B24] Neuschwander-Tetri B. A. Loomba R. Sanyal A. J. Lavine J. E. Van Natta M. L. Abdelmalek M. F. (2015). Farnesoid X nuclear receptor ligand obeticholic acid for non-cirrhotic, non-alcoholic steatohepatitis (FLINT): a multicentre, randomised, placebo-controlled trial. Lancet 385 (9972), 956–965. 10.1016/s0140-6736(14)61933-4 25468160 PMC4447192

[B25] Page M. J. McKenzie J. E. Bossuyt P. M. Boutron I. Hoffmann T. C. Mulrow C. D. (2021). The PRISMA 2020 statement: an updated guideline for reporting systematic reviews. Bmj 372, n71. 10.1136/bmj.n71 33782057 PMC8005924

[B26] Pothula R. V. K. Kurapati S. Balineni S. K. Gogineni N. T. (2022). A blend of *Sphaeranthus indicus* flower head and *Terminalia chebula* fruit extracts reduces fatty liver and improves liver function in non-alcoholic, overweight adults. Funct. Foods Health Disease 12 (7), 361–379. 10.31989/ffhd.v12i7.958

[B27] Rong L. Zou J. Ran W. Qi X. Chen Y. Cui H. (2022). Advancements in the treatment of non-alcoholic fatty liver disease (NAFLD). Front. Endocrinol. (Lausanne) 13, 1087260. 10.3389/fendo.2022.1087260 36726464 PMC9884828

[B28] Sanyal A. J. Chalasani N. Kowdley K. V. McCullough A. Diehl A. M. Bass N. M. (2010). Pioglitazone, vitamin E, or placebo for nonalcoholic steatohepatitis. N. Engl. J. Med. 362 (18), 1675–1685. 10.1056/NEJMoa0907929 20427778 PMC2928471

[B29] Sayiner M. Stepanova M. Pham H. Noor B. Walters M. Younossi Z. M. (2016). Assessment of health utilities and quality of life in patients with non-alcoholic fatty liver disease. BMJ Open Gastroenterol. 3 (1), e000106. 10.1136/bmjgast-2016-000106 27648297 PMC5013331

[B30] Sterne J. A. C. Savović J. Page M. J. Elbers R. G. Blencowe N. S. Boutron I. (2019). RoB 2: a revised tool for assessing risk of bias in randomised trials. Bmj 366, l4898. 10.1136/bmj.l4898 31462531

[B31] Takahashi H. Kessoku T. Kawanaka M. Nonaka M. Hyogo H. Fujii H. (2022). Ipragliflozin improves the hepatic outcomes of patients with diabetes with NAFLD. Hepatol. Commun. 6 (1), 120–132. 10.1002/hep4.1696 34558835 PMC8710792

[B32] Tapper E. B. Lai M. (2016). Weight loss results in significant improvements in quality of life for patients with nonalcoholic fatty liver disease: a prospective cohort study. Hepatology 63 (4), 1184–1189. 10.1002/hep.28416 26698379 PMC4805484

[B33] The World Health Organization Quality of Life assessment (WHOQOL) (1995). Position paper from the World Health Organization. Soc. Sci. Med. 41 (10), 1403–1409. 10.1016/0277-9536(95)00112-k 8560308

[B34] Vogli S. Naska A. Marinos G. Kasdagli M. I. Orfanos P. (2023). The effect of vitamin E supplementation on serum aminotransferases in non-alcoholic fatty liver disease (NAFLD): a systematic review and meta-analysis. Nutrients 15 (17), 3733. 10.3390/nu15173733 37686767 PMC10490270

[B35] Wah Kheong C. Nik Mustapha N. R. Mahadeva S. (2017). A randomized trial of silymarin for the treatment of nonalcoholic steatohepatitis. Clin. Gastroenterol. Hepatol. 15 (12), 1940–9.e8. 10.1016/j.cgh.2017.04.016 28419855

[B36] Wang Y. Yi H. Sun W. Yu H. Tao W. Yu X. (2024). Comparative efficacy of drug interventions on NAFLD over 24 weeks: a traditional and network meta-analysis of randomized controlled trials. Drugs 84 (4), 425–439. 10.1007/s40265-024-02015-6 38478331

[B37] Ware J. E. Jr. Sherbourne C. D. (1992). The MOS 36-item short-form health survey (SF-36). I. Conceptual framework and item selection. Med. Care 30 (6), 473–483. 10.1097/00005650-199206000-00002 1593914

[B38] Younossi Z. M. Stepanova M. Noureddin M. Kowdley K. V. Strasser S. I. Kohli A. (2021). Improvements of fibrosis and disease activity are associated with improvement of patient-reported outcomes in patients with advanced fibrosis due to nonalcoholic steatohepatitis. Hepatol. Commun. 5 (7), 1201–1211. 10.1002/hep4.1710 34278169 PMC8279457

[B39] Younossi Z. M. Stepanova M. Taub R. A. Barbone J. M. Harrison S. A. (2022a). Hepatic fat reduction due to resmetirom in patients with Nonalcoholic steatohepatitis is associated with improvement of quality of life. Clin. Gastroenterol. Hepatol. 20 (6), 1354–61.e7. 10.1016/j.cgh.2021.07.039 34329774

[B40] Younossi Z. M. Stepanova M. Nader F. Loomba R. Anstee Q. M. Ratziu V. (2022b). Obeticholic acid impact on quality of life in patients with nonalcoholic steatohepatitis: REGENERATE 18-Month interim analysis. Clin. Gastroenterol. Hepatol. 20 (9), 2050–8.e12. 10.1016/j.cgh.2021.07.020 34274514

[B41] Younossi Z. M. Paik J. M. Stepanova M. Ong J. Alqahtani S. Henry L. (2024). Clinical profiles and mortality rates are similar for metabolic dysfunction-associated steatotic liver disease and non-alcoholic fatty liver disease. J. Hepatology 80 (5), 694–701. 10.1016/j.jhep.2024.01.014 38286339

[B42] Younossi Z. M. Stepanova M. Racila A. Henry L. Labriola D. Taub R. (2025). Health-related quality of life (HRQL) assessments in a 52-week, double-blind, randomized, placebo-controlled phase III study of resmetirom (MGL-3196) in patients with metabolic dysfunction-associated steatohepatitis (MASH) and fibrosis. Hepatology 81 (4), 1318–1327. 10.1097/hep.0000000000001084 39250515

