# Supplementary material for: The role of pharmacotherapy in improving quality of life among patients with MAFLD: a systematic review and meta-analysis of randomized trials
Source: Front Pharmacol. 2026 Oct 19;17:1788802. doi: 10.3389/fphar.2026.1788802 (PMC13725905; doi:10.3389/fphar.2026.1788802)
Supplement: Supplementary file 1 [file Supplementaryfile1.docx]

**Content**

[Table S1. Search Strategy	2](#_Toc165670811)

[Table S2. GRADE working group grades of evidence	4](#_Toc165670812)

[Figure S1-S9 Forest plot for Short Form 36 Health Survey (SF-36)	7](#_Toc165670813)

[Figure S10-S15 Forest plot forChronic Liver Disease Questionnaire (CLDQ)	16](#_Toc165670814)

[Figure S16-19 Subgroup analysis for SF-36	22](#_Toc165670815)

Figure S20-26 Sensitivity analysis.................................................................................................................26

Table S1: Search Strategy

| database | # | Query | Results |
| --- | --- | --- | --- |
| Pubmed | 1  2  3  4  5 | "Non-alcoholic Fatty Liver Disease"[Mesh]  Non alcoholic Fatty Liver Disease[Title/Abstract] OR NAFLD[Title/Abstract] OR Nonalcoholic Fatty Liver Disease[Title/Abstract] OR MAFLD[Title/Abstract]OR Metabolic Dysfunction-associated Steatotic Liver Disease[Title/Abstract]OR Fatty Liver, Nonalcoholic[Title/Abstract] OR Fatty Livers, Nonalcoholic[Title/Abstract] OR Liver, Nonalcoholic Fatty[Title/Abstract] OR Livers, Nonalcoholic Fatty[Title/Abstract] OR Nonalcoholic Fatty Liver[Title/Abstract] OR Nonalcoholic Fatty Livers[Title/Abstract] OR Nonalcoholic Steatohepatitis[Title/Abstract] OR Nonalcoholic Steatohepatitides[Title/Abstract] OR Steatohepatitides, Nonalcoholic[Title/Abstract] OR Steatohepatitis, Nonalcoholic[Title/Abstract]  "Quality of Life"[Mesh]  Life Quality OR Health-Related Quality Of Life OR Health Related Quality Of Life OR HRQOL OR Quality of Life  (#1 OR #2) AND (#3 OR #4) | 29739  52971  313120  670740  724 |
| Embase | 1  2  3  4  5 | 'nonalcoholic fatty liver'/exp  'nafld (nonalcoholic fatty liver disease)':ab,ti OR 'non alcoholic fatty liver disease':ab,ti OR 'non alcoholic hepato-steatosis':ab,ti OR 'non alcoholic hepatosteatosis':ab,ti OR 'non alcoholic liver steatosis':ab,ti OR 'non alcoholic steatotic hepatopathy':ab,ti OR 'non-alcoholic fatty liver':ab,ti OR 'non-alcoholic fatty liver disease':ab,ti OR 'non-alcoholic fld':ab,ti OR 'non-alcoholic hepatic steatosis':ab,ti OR 'nonalcoholic fatty liver disease':ab,ti OR 'nonalcoholic fld':ab,ti OR 'nonalcoholic hepatic steatosis':ab,ti OR 'nonalcoholic hepatosteatosis':ab,ti OR 'nonalcoholic liver steatosis':ab,ti OR 'nonalcoholic fatty liver':ab,ti OR 'mafld (metabolic dysfunction-associated steatotic liver disease)'  'quality of life'/exp  'health related quality of life':ab,ti OR 'hrql':ab,ti OR 'life quality':ab,ti OR 'quality of life':ab,ti  (#1 OR #2) AND (#3 OR #4) | 85143  57991  846641  755935  1857 |
| Cochrane Library | 1  2  3  4  5 | MeSH descriptor: [Non-alcoholic Fatty Liver Disease] explode all trees  (Non alcoholic Fatty Liver Disease):ti,ab,kw OR (Nonalcoholic Fatty Livers):ti,ab,kw OR (NAFLD):ti,ab,kw OR (MAFLD):ti,ab,kw OR (Metabolic Dysfunction-associated Steatotic Liver ):ti,ab,kw (Liver, Nonalcoholic Fatty):ti,ab,kw OR (Fatty Livers, Nonalcoholic):ti,ab,kw(Nonalcoholic Fatty Liver):ti,ab,kw OR (Livers, Nonalcoholic Fatty):ti,ab,kw OR (Fatty Liver, Nonalcoholic):ti,ab,kw OR (Nonalcoholic Fatty Liver Disease):ti,ab,kw OR (Nonalcoholic Steatohepatitis):ti,ab,kw(Steatohepatitides, Nonalcoholic):ti,ab,kw OR (Nonalcoholic Steatohepatitides):ti,ab,kw OR (Steatohepatitis, Nonalcoholic):ti,ab,kw OR (Non-alcoholic Fatty Liver Disease):ti,ab,kw  MeSH descriptor: [Quality of Life] explode all trees  (Life Quality) OR (Health Related Quality Of Life) OR (Health-Related Quality Of Life) OR (HRQOL) OR (Quality of Life)  (#1 OR #2) AND (#3 OR #4) | 1984  5767  45907  199856  262 |
| Web of Science | 1 | (TS=(Non alcoholic Fatty Liver Disease OR NAFLD OR Nonalcoholic Fatty Liver Disease OR MAFLD OR Metabolic Dysfunction-associated Steatotic Liver Disease OR Fatty Liver, Nonalcoholic OR Fatty Livers, Nonalcoholic OR Liver, Nonalcoholic Fatty OR Livers, Nonalcoholic Fatty OR Nonalcoholic Fatty Liver OR Nonalcoholic Fatty Livers OR Nonalcoholic Steatohepatitis OR Nonalcoholic Steatohepatitides OR Steatohepatitides, Nonalcoholic OR Steatohepatitis, Nonalcoholic OR Non-alcoholic Fatty Liver Disease)) AND ALL=(Life Quality OR Health-Related Quality Of Life OR Health Related Quality Of Life OR HRQOL OR Quality of Life) | 894 |

Table S2: GRADE working group grades of evidence

| Outcomes | | Quality assessment | | | | | | | | | No of patients | | | | | Effect | | | Quality | |
| --- | --- | --- | --- | --- | --- | --- | --- | --- | --- | --- | --- | --- | --- | --- | --- | --- | --- | --- | --- | --- |
|  | Studies  (n) | | Design | | Risk of bias | | Inconsistency | Indirectness | Imprecision | Other  considerations | EG | | CG | | | Relative  (95%CI) | Absolute | | |  |
| Physical component summary(SF-36) | | 7 | | RCT | | Serious | No Serious | No Serious | No Serious | No Serious | | 445 | | 339 | SMD 0.19 (0.05 to 0.34) | | | Small | | ⊕⊕⊕⊝  moderate |
| Mental component  summary(SF-36) | | 7 | | RCT | | Serious | No Serious | No Serious | No Serious | No Serious | | 445 | | 339 | SMD -0.08 (-0.3 to 0.13) | | | Not estimable from available data | | ⊕⊕⊕⊝  moderate |
| Total score (CLDQ) | | 4 | | RCT | | Serious | No Serious | No Serious | No Serious | No Serious | | 1375 | | 721 | SMD -0.05 (-0.14 to 0.04) | | | Not estimable from available data | | ⊕⊕⊕⊝  moderate |
| Physical functioning(SF-36) | | 3 | | RCT | | Serious | No Serious | Serious | Serious | No Serious | | 126 | | 88 | SMD -0.05 (-0.14 to 0.04) | | | Not estimable from available data | | ⊕⊝⊝⊝  very low |
| Physical role functioning(SF-36) | | 3 | | RCT | | Serious | No Serious | Serious | Serious | No Serious | | 156 | | 88 | SMD 0.29 (-0.15 to 0.73) | | | Not estimable from available data | | ⊕⊝⊝⊝  very low |
| Bodily pain(SF-36) | | 3 | | RCT | | Serious | No Serious | Serious | Serious | No Serious | | 156 | | 88 | SMD 0.13 (-0.16 to 0.43) | | | Not estimable from available data | | ⊕⊝⊝⊝  very low |
| General health perceptions(SF-36) | | 3 | | RCT | | Serious | No Serious | Serious | Serious | No Serious | | 126 | | 88 | SMD 0.97 (-0.37 to 2.32) | | | Not estimable from available data | | ⊕⊝⊝⊝  very low |
| Vitality(SF-36) | | 3 | | RCT | | Serious | No Serious | Serious | Serious | No Serious | | 156 | | 88 | SMD 0.26 (-0.01 to 0.52) | | | Not estimable from available data | | ⊕⊝⊝⊝  very low |
| Social functioning(SF-36) | | 3 | | RCT | | Serious | No Serious | Serious | Serious | No Serious | | 156 | | 88 | SMD 0.17 (-0.1 to 0.43) | | | Not estimable from available data | | ⊕⊝⊝⊝  very low |
| Emotional role functioning(SF-36) | | 3 | | RCT | | Serious | No Serious | Serious | Serious | No Serious | | 156 | | 88 | SMD 0.05 (-0.27 to 0.37) | | | Not estimable from available data | | ⊕⊝⊝⊝  very low |
| Mental health(SF-36) | | 3 | | RCT | | Serious | No Serious | Serious | Serious | No Serious | | 156 | | 88 | SMD 0.14 (-0.13 to 0.4) | | | Not estimable from available data | | ⊕⊝⊝⊝  very low |
| Worry(CLDQ) | | 3 | | RCT | | Serious | No Serious | Serious | Serious | No Serious | | 1355 | | 700 | SMD 0 (-0.22 to 0.22) | | | Not estimable from available data | | ⊕⊝⊝⊝  very low |
| Fatigue symptoms(CLDQ) | | 3 | | RCT | | Serious | No Serious | Serious | Serious | No Serious | | 1355 | | 700 | SMD 0.01 (-0.19 to 0.21) | | | Not estimable from available data | | ⊕⊝⊝⊝  very low |
| Systemic symptoms(CLDQ) | | 3 | | RCT | | Serious | No Serious | Serious | Serious | No Serious | | 1355 | | 700 | SMD -0.06 (-0.16 to 0.03) | | | Not estimable from available data | | ⊕⊝⊝⊝  very low |
| Activity(CLDQ) | | 3 | | RCT | | Serious | No Serious | Serious | Serious | No Serious | | 1355 | | 700 | SMD 0.25 (-0.13 to 0.62) | | | Not estimable from available data | | ⊕⊝⊝⊝  very low |
| Emotional functioning(CLDQ) | | 3 | | RCT | | Serious | No Serious | Serious | Serious | No Serious | | 1355 | | 700 | SMD -0.04 (-0.14 to 0.05) | | | Not estimable from available data | | ⊕⊝⊝⊝  very low |
| Abdominal symptoms(CLDQ) | | 3 | | RCT | | Serious | No Serious | Serious | Serious | No Serious | | 1355 | | 700 | SMD 0 (-0.09 to 0.09) | | | Not estimable from available data | | ⊕⊝⊝⊝  very low |

Figure S1 Forest plot for overall Physical functioning(SF-36)

Figure S2 Forest plot for overall Physical role functioning(SF-36)

Figure S3 Forest plot for overall Bodily pain(SF-36)

Figure S4 Forest plot for overall General health perceptions(SF-36)

Figure S5 Forest plot for overall Vitality(SF-36)

Figure S6 Forest plot for overall Social functioning(SF-36)

Figure S7 Forest plot for overall Emotional role functioning(SF-36)

Figure S8 Forest plot for overall Mental health(SF-36)

Figure S9 Forest plot for overall Mental health(SF-36)

Figure S10 Forest plot for overall Worry(CLDQ)

Figure S11 Forest plot for overall Fatigue symptoms(CLDQ)

Figure S12 Forest plot for overall Systemic symptoms(CLDQ)

 Figure S13 Forest plot for overall Activity(CLDQ)

 Figure S14 Forest plot for overall Emotional functioning(CLDQ)

Figure S15 Forest plot for overall Abdominal symptoms(CLDQ)

Figure S16 Subgroup analysis for Physical component summary(SF-36)a

Figure S17 Subgroup analysis for Physical component summary(SF-36)b

Figure S18 Subgroup analysis for Mental component summary(SF-36)a

 Figure S19 Subgroup analysis for Mental component summary(SF-36)b

Figure S20 Sensitivity analysis(a)

Figure S21 Sensitivity analysis(b)

Figure S22 Sensitivity analysis(c)

Figure S23 Sensitivity analysis(d)

Figure S24 Sensitivity analysis(e)

 Figure S25 Sensitivity analysis(f)

Figure S26 Sensitivity analysis(g)
